# Sparse Representations-Based Super-Resolution of Key-Frames Extracted from Frames-Sequences Generated by a Visual Sensor Network

**DOI:** 10.3390/s140203652

**Published:** 2014-02-21

**Authors:** Muhammad Sajjad, Irfan Mehmood, Sung Wook Baik

**Affiliations:** College of Electronics and Information Engineering, Sejong University, Seoul 143-747, Korea; E-Mails: sajjad@sju.ac.kr (M.S.); irfanmehmood@sju.ac.kr (I.M.)

**Keywords:** visual sensor, super-resolution, redundant dictionary, matching pursuit

## Abstract

Visual sensor networks (VSNs) usually generate a low-resolution (LR) frame-sequence due to energy and processing constraints. These LR-frames are not very appropriate for use in certain surveillance applications. It is very important to enhance the resolution of the captured LR-frames using resolution enhancement schemes. In this paper, an effective framework for a super-resolution (SR) scheme is proposed that enhances the resolution of LR key-frames extracted from frame-sequences captured by visual-sensors. In a VSN, a visual processing hub (VPH) collects a huge amount of visual data from camera sensors. In the proposed framework, at the VPH, key-frames are extracted using our recent key-frame extraction technique and are streamed to the base station (BS) after compression. A novel effective SR scheme is applied at BS to produce a high-resolution (HR) output from the received key-frames. The proposed SR scheme uses optimized orthogonal matching pursuit (OOMP) for sparse-representation recovery in SR. OOMP does better in terms of detecting true sparsity than orthogonal matching pursuit (OMP). This property of the OOMP helps produce a HR image which is closer to the original image. The K-SVD dictionary learning procedure is incorporated for dictionary learning. Batch-OMP improves the dictionary learning process by removing the limitation in handling a large set of observed signals. Experimental results validate the effectiveness of the proposed scheme and show its superiority over other state-of-the-art schemes.

## 1. Introduction

A visual sensor network (VSN) is a distributed wireless system that interacts with the physical environment by observing it through a visual sensor. It consists of a group of camera nodes, each equipped with a low-power, embedded processor, energy source, image sensor, and some type of transceiver for communication. These nodes must have the capability of communication with a visual processing hub (VPH), where the visual-data is collected and some image processing operations are applied before sending it to the base station (BS). Embedded sensing devices in VSNs are constrained in terms of battery, memory, processing capability, and achievable date-rate. VSNs offer a wide range of applications. Visual sensors are used to enhance the existing surveillance system against crime and terrorist attacks. VSNs can extend the ability of law-enforcement agencies to monitor public areas, private properties, public events, and borders. They can gather and record potentially relevant activities (for example, traffic violations or thefts) and make video frames available for future analysis. VSNs can also be used for personal and health care activities. For example, they can monitor the behavior of elderly people as a means to identify causes of illnesses such as dementia [1,2]. Other applications of VSN include gaming, environmental, industrial, and virtual reality.

VSNs offer new opportunities for many promising applications compared to other sensor networks. VSNs pose new challenges that are not fully addressed by current research on wireless sensor networks (WSNs). Visual sensors collect huge amounts of data compared to scalar sensors. This collected visual data should be processed at the VPH and only relevant data is transmitted to BS. Most of the researchers in the area of VSNs agree that transmitting all the visual data to BS is impractical because of two major constraints: energy and bandwidth [3]. For this purpose, various schemes based on key-frame extraction have been developed to produce summaries of the videos captured by camera sensors [4–6]. In these techniques, summaries are generated by extracting the most relevant frames from the frame sequences. The authors in [4] used a multi-dimensional-based clustering technique to automatically generate high-quality video summaries most suitable for wireless and mobile communication. Chen *et al.* [7] extracted key-frames of basketball video using automatic scene analysis and camera viewpoint selection. In [8], the authors incorporated casting text along with the video analysis algorithms for the extraction of key-frames from sport videos. For a detailed review of the existing techniques using key-frame extraction techniques, the readers are referred to the survey published in [5]. In addition, image sensors consume more energy than traditional sensor nodes and streaming of captured images also requires a large segment of bandwidth that causes a huge amount of power consumption. It is necessary to compress the visual data and only send the most relevant compressed data to BS. Hence, visual sensors used in VSNs are usually low-quality, having low precision and resolution. Therefore, the images captured by the visual sensors are not very appropriate for use in various applications. A high-resolution (HR) display (for example an LCD) is unable to display the streaming of captured images that are low-resolution (LR). However, resolution enhancement is needed in many applications (surveillance, health-care, traffic monitoring and enforcement) for HR displays. Analysis and diagnosis are very difficult from LR images in medical imaging. It is also challenging to analyze the scene from the images captured by surveillance cameras for a particular purpose. In many surveillance video applications, it is of great interest to recognize an object such as number, words, or labels that often occupy a small portion of a LR noisy video. In these circumstances, HR images containing clear edges of the object are very useful and important [9,10]. Moreover, a specific part of an LR image that is selected as a region-of-interest (ROI) often needs to be in HR for analysis because it is very difficult to achieve a particular required goal from the LR images. Two common approaches are used to get an HR image from its corresponding LR image. In the first approach, large numbers of high-quality and expensive visual sensors are used to capture a particular scene in better quality and HR. The size of each pixel in such frames is bigger than the pixel in the frame captured by the low-quality sensor, which in turn increases the size of the generated frame. But, it causes high energy consumption due to the fact that: (1) HR images need extensive computation for initial processing at the camera node such as filtering, *etc*. and (2) high-bandwidth is needed to transmit high-quality HR frames to the VPH and then to the BS [3,11]. It is also important to mention that high-quality frames are not usually required at the camera end but are required at the BS for scene analysis. Therefore, this is not a feasible solution where battery and bandwidth are the major constraints of the underlying VSN. Hence, super-resolution (SR) algorithms (i.e., the second approach) that reconstruct an HR image from the corresponding LR image captured by the visual sensor node (i.e., low-quality visual sensors) have been widely researched in the last two decades. SR algorithms are usually implemented on work-stations where energy consumption is not a problem. In this approach, frames of low-quality are transmitted from visual sensors to VPH and then to BS and as a result consume less energy and bandwidth than the first approach [3].

SR techniques overcome the inherent resolution limitations by computing the missing or unknown information at the pixel level. Bicubic, bilinear and nearest-neighbor are more basic non-adaptive interpolation techniques that are used to compute a HR image from its corresponding LR image. These interpolation methods cannot recover the missing high frequency components and often blur the discontinuities of the HR image. In order to minimize the aliasing effects of the non-adaptive interpolation schemes, various non-linear adaptive interpolation algorithms have been developed. Jurio *et al.* [12] have proposed an image SR technique using interval information that associates each pixel with an interval obtained by weighted aggregation of the pixels in its neighborhood. Shi *et al.* [13] proposed an edge-directed adaptive interpolation SR algorithm for VSNs. It estimates the value of the unknown pixel using a weighted average of its neighboring pixels (that is, it gives more weight to the nearer pixels and pixels belonging to the direction with smaller variation). The authors did not specify the place of implementation in VSNs (that is, camera node, VPH or BS). In our previous work, we proposed an adaptive interpolation scheme based on a multi-kernel approach for image super-resolution [14]. The main drawback of these algorithms is that they cannot conduct SR and denoising simultaneously. They also magnify the noisy pixels and cannot recover the actual information. In order to reduce the bad artifacts of the adaptive and non-adaptive schemes, various techniques (that is, based on the inverse problem, machine learning, sparse representation, and using motion abilities of the camera sensor node including pan, tilt and zoom) are incorporated. Bose and Boo [15] proposed an SR algorithm to fuse multiple shifted and degraded images acquired by multi-sensors. Caner *et al.* [9] and Shechtman *et al.* [16] presented the problem of SR recovery for constructing a video sequence of high space-time resolution by combining information from multiple low-resolution video sequences of the same dynamic scene. Both algorithms are resource hungry in terms of energy and bandwidth because they conduct onboard processing and then transmit SR video sequences onward for further processing. In [17], the authors developed a blind image restoration algorithm to reconstruct a high resolution color image from multiple, low resolution, degraded and noisy images captured by a thin observation module by bound optics. The proposed algorithm in [17] is very specific and cannot be extended to other visual sensors. It is also not a good option to implement schemes having rich processing on the camera node because such schemes utilize a significant amount of power. Kansal *et al.* [18] added motion abilities such as pan, tilt, and zooming to camera sensors to provide virtual HR in VSNs. The mechanism of controlled motion held in avoiding obstacles and camera overlap provides an image which has an acceptable level of details. Tezcan *et al.* [19] also proposed an automated orientation selection algorithm that finds the most beneficial pose of the visual sensors to maximize the multimedia coverage with occlusion free view-points. Both schemes have many mechanical operations that require more power and time and lead the VSN to die soon. The authors in [20] presented an edge-preserving maximum *a posteriori* (MAP) estimation-based SR algorithm using a weighted directional Markov image prior model for a ROI from more than one LR surveillance image. It incorporates a conjugate gradient (CG) to improve the computational efficiency of the algorithm. The authors in [20] did not specify the place of implementation. However, the onboard processing of such resource-expensive algorithms causes too much energy consumption.

To cope with these constraints, this paper presents an effective SR framework for summarized frames generated from the frame-sequences captured by the visual-sensors. VPH receives video sequences in large amounts from visual-sensors. In order to minimize the bandwidth consumptions and stream only the relevant and non-redundant frames to BS, key-frames are generated using our recent video summarization technique [21]. Compression is also applied to the key-frames at the VPH. The proposed SR scheme computes the HR version of the received LR compressed key-frames using an overcomplete dictionary. The dictionary uses a K-SVD dictionary learning procedure augmented by incorporating batch orthogonal matching pursuit (batch-OMP) for seeking sparse coefficients. Batch-OMP is a good option to handle a large set of training signals than a simple orthogonal matching pursuit (OMP). In SR, we use optimized orthogonal matching pursuit (OOMP) because it is more robust in terms of detecting true sparsity than OMP [22]. A variety of experiments were conducted to prove the effectiveness and validity of the proposed scheme over other state-of-the-art schemes.

The rest of the paper is organized as follows: In Section 2, the related work is presented. Section 3 discusses the proposed framework in detail. Experimental results are described in Section 4 and Section 5 briefly describes a prospective example based study related to VSN. The paper is concluded in Section 6.

## 2. Related Work

In this section, we cover various SR techniques ranging from simple interpolation to complex machine learning algorithms. SR schemes are broadly categorized in three types: (1) interpolation based SR algorithms; (2) inverse problem based SR techniques; and (3) machine learning based SR schemes. In each category, some algorithms have been designed keeping in mind the specific requirements of the VSNs, i.e., domain-specific, and some have been proposed while considering the general requirements of SR (that is, not specifically designed for VSNs).

There are two types of interpolation schemes: (1) non-adaptive interpolation; (2) adaptive interpolation. In non-adaptive interpolation, the nearest neighbor (NN) is the most simple interpolation scheme, which considers only one pixel: the closest one to the interpolated point. Bilinear interpolation computes the unknown pixel by taking the average of the closest 2 × 2 known-pixels in the neighborhood. Bicubic interpolation scheme goes one step beyond bilinear scheme by considering the closest 4 × 4 neighborhood of known pixels. These schemes are non-adaptive and have very low-time complexity, but they produce bad artifacts (for example, edge blurring, blocking effects or stair cases) in SR images. Many algorithms using adaptive-interpolation have been proposed to reduce the bad-artifacts of the non-adaptive interpolation. These limitations are somewhat reduced by replacing them with adaptive interpolation schemes [12,14,23].

In the second category of SR approaches, the SR task is cast as an inverse problem to construct an HR frame of better quality by fusing multiple LR frames in the VSN based on prior knowledge of the observation model. Caner *et al.* [9] proposed an SR technique that reconstructed ROI from more than one LR view of the scene recorded by multiple visual sensor nodes. In this method, a view is registered for accurate spatiotemporal alignment of multiple video sequences and then projects onto convex sets. Two factors: (1) the accuracy of multiple-view registration and (2) data acquisition from the correct visual sensor node are very important. Shechtman *et al.* [16] followed the same approach by constructing the HR output from multiple LR video sequences of the same dynamic scene. Simultaneous denoising and SR in space-time are the main features of this algorithm. In some SR techniques, a hybrid approach has been incorporated that combines the benefit of both inverse problem and machine learning algorithms. In [24], the authors presented an efficient multi-frame super-resolution mosaicking algorithm. SIFT is used for features matching between frames to estimate the homograph. Next, separate frames are registered and the overlapping region is extracted. Sparse representations are determined using a hybrid regularization method and then applied to the individual sub-frames to compute the HR version. Zhang *et al.* [20] developed an edge-preserving maximum *a posteriori* (MAP)-based SR technique using a weighted directional Markov image prior model from multiple LR surveillance images. In [17], the authors developed an FFT-based blind image restoration technique to reconstruct an HR color image from multiple, LR, degraded and noisy images captured by thin observation module by bounded optics. This spectral based color image restoration method computes average of all LR captured images by making independent the color channels of each other globally. In [25], a Bayesian-based SR scheme was presented that computes wavelet coefficients of the target image from: (1) multiple images observed in VSN and (2) a prior image that is imposed by the component exponential mixture model. In these algorithms, the result is not photo-realistic because it is mostly dependent on the local image singularities.

Mechanical operations of the camera node can be incorporated for enhancing the quality of HR output. In [18] how controlled motion can help in providing HR sensing in VSN was discussed. The authors added motion abilities such as pan, tilt, and zoom to visual sensor to provide an HR view of the scene. They stated that the mechanism of controlled motion helps in avoiding obstacles and camera overlap and provides an image which has an acceptable level of details for various image processing operations. In [19], an automated orientation selection algorithm was proposed to find the most beneficial pose of the visual sensor node to maximize the scene coverage with occlusion free viewpoints. It reduces the effect of occlusion in the scene by increasing the resolution and the cumulative quality of the information sensed from the ROI. Aghdasi *et al.* [26] proposed a two-tier network structure and camera selection algorithm that acquire an HR image with a wide FOV by using image stitching in wireless multimedia sensor networks (WMSNs). The authors claim that using image stitching in VSNs allows: (1) the minimization of energy dissipation and (2) the acquisition of the HR image that satisfy the requirements of many VSN based applications. In [27] a multi-resolution (MR) scheme with steerable focus, e-Fovea, which provides peripheral vision with a steerable fovea that is in HR was presented. It is a hybrid dual camera system having MR on both input/output sides which enables users to focus on ROI in very HR and be aware of the peripheral information in LR simultaneously. In this method, e-Fovea is not fully simulated with variable zoom. The selection of ROI is also determined by humans via mouse click. Moreover it is for a single user and needs extra resources to get the required result. These SR schemes have many mechanical operations in the form of visual-sensor motion and require heavy processing that causes more energy consumption and leads the WMSN to die soon. They produce the HR frames at a sensor level that causes high energy and bandwidth consumption at the very initial stages.

Various machine learning-based algorithms were developed to address the SR problem by considering its general requirements. These algorithms attempt to capture the co-occurrence prior between LR and HR image patches. Yang *et al.* [28] approached the SR problem by assuming the LR image as a downsampled version of the HR image, whose patches have a sparse representation with respect to an over-complete dictionary. Yang *et al.* further improved their work in [29] by using a joint dictionary learning method for two different feature spaces (LR and HR) tied by a mapping function which determines sparse representation for each LR patch. They employed *l_1_*-regularized linear regression for sparse representation recovery. As it comes in basis pursuit (BP) category, and it is generally believed that basis pursuit algorithms can produce more accurate solutions than the orthogonal matching pursuit (OMP), usually BP algorithms need more computational effort [30]. The recovered representations were then used to generate an HR output. For dictionary learning, Yang *et al.* [29] incorporated the work of Lee *et al.* [31]. Further, Zeyde *et al.* [32] modified the work of Yang *et al.* [29] by incorporating numerical shortcuts and dimensionality reduction via Principal Component Analysis (PCA) to make it more efficient. The authors used the same approach in terms of dictionary learning to enforce the sparse representations similarity to construct the HR out, but the authors incorporated the K-SVD approach [33] for dictionary learning. Zeyde *et al.* [32] used OMP to recover the vector of sparse representations in both cases (that is, SR and dictionary learning).

## 3. Framework of the Proposed System

**Figure 1.**
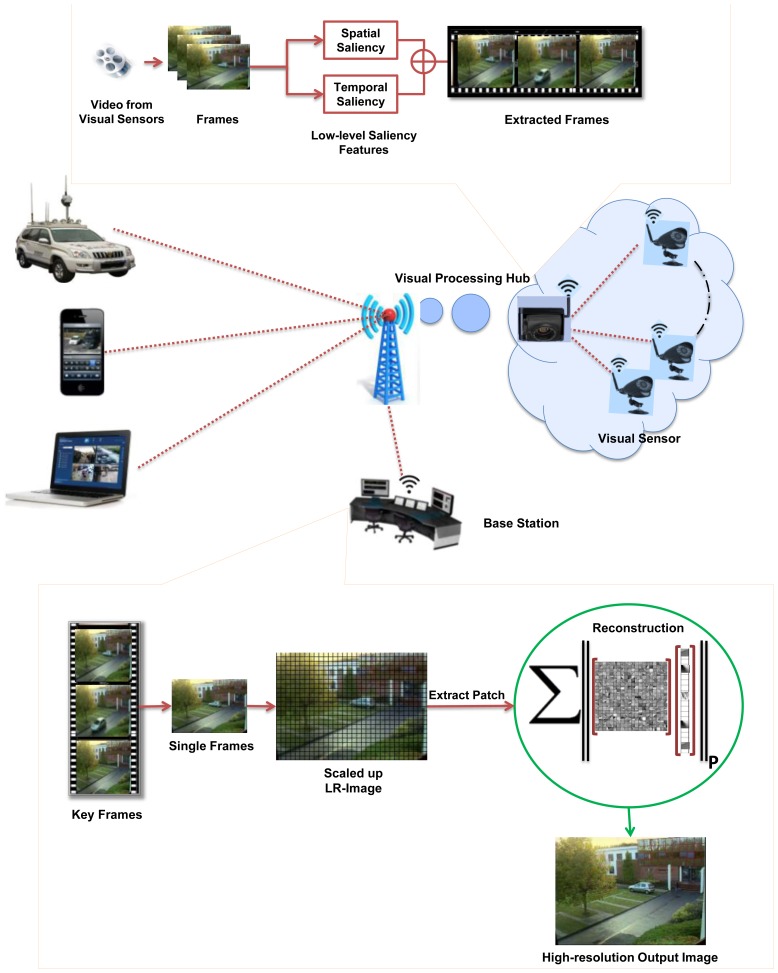
Framework of the proposed system.

Camera sensor nodes collect data in the form of LR video sequences and send it to a VPH for further processing. VPHs have comparatively larger computational resources than visual-sensor nodes and are suitable for some initial image processing operations such as key-frame extraction and compression. It summarizes the large amount of collected visual data efficiently and sends it to the BS. The BS is a work-station that provides an ideal environment for operations needing high computational efforts and energy. The frames sequence received at a BS have low precision and resolution and are not appropriate for use in various applications. Therefore, an effective image SR module of the proposed system is applied on received LR visual data at BS. Figure 1 shows the framework of the proposed system.

### 3.1. Summarization of Video Sequences Received from Visual Sensors

VPHs collect visual data in a large amount from visual sensor nodes. It is generally assumed that streaming all the visual data is impractical due to the severe energy and bandwidth constraints of VSNs [11]. The bandwidth and energy cost of transmitting video data to BS is more expensive, because the BS is usually away from VPHs. For this purpose, various methods (such as compression and video summarization) have been applied to reduce the size of the collected visual data at VPH and only relevant data is transmitted to BS [4,34,35]. To keep in view the energy and bandwidth constraint, we have used our recent video summarization scheme [21] based on an efficient visual attention model that extracts more relevant key frames from videos by minimizing the redundancy. The computational cost of our recent video summarization approach is low because it uses a temporal-gradient-based dynamic visual-saliency detection procedure instead of the traditional computationally rich optical-flow techniques. Low-level saliency features such as spatial and temporal attention are used to extract more relevant key-frames from the video. Suppose VPH receives video V={v_1_, …, v*_n_*} from visual sensors node consisting of *n* frames. For each frame v*_i_*, the spatial attention value is calculated as:

(1) $${\text{V}}_{\text{s}}=f_{\text{s}}(\text{V})$$

where *f_S_* is a function computes the spatial attention value for each frame v*_i_* in the video V. The concept of an image descriptor called image signature [36] is used to compute the visual saliency, on which the visual attention model of the proposed framework is based. The image signature can be used to approximate the foreground of each frame v*_i_* in the video V because the foreground of an image (i.e., frame v*_i_*) is visually more noticeable than its background. V_S_ is a vector contains spatial attention values for each frame in the range 0 to1. The frame is considered salient, if its spatial attention value is close to one and *vice versa*.

According to human perception, in video, object motion relative to each other is more important. Therefore, motion is an important constituent to model human attention for videos. The concept of temporal gradient is employed to obtain motion information quickly from video sequences. The temporal gradients measure the change in pixel values across the frames and thus compute the motion component of visual attention. Suppose *f*_
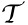
_ is a function that computes the visual attention value for each frame v*_i_* in the video sequence V obtained from the visual sensor node:

(2) $${\text{V}}_{\text{T}}=f_{T}(\text{V})$$

where V_T_ is a vector containing temporal attention values for each frame v*_i_* in the video sequence V in 0 to1. A value close to one indicates that the frame is salient, otherwise the frame will be considered a non-salient frame. The next step is the fusion of V_S_ and V_T_ to get the final attention value for key-frame extraction. A non-linear fusion scheme has been incorporated for fusion in our work that replicates human visual perception [37]. This is because theories related to human visual perception claim that the motion component is more important compared to the static attention clues. The fusion scheme used in the proposed framework combine the benefits of both spatial and temporal attention:

(3) $$\text{Ṽ}=ℱ(\text{V},{\text{V}}_{\text{S}},{\text{V}}_{\text{T}})$$

where ℱ is a function that fuses V_S_ and V_T_ and return a summarized video Ṽ = {ṽ_1_,…,ṽ_m_} consisted of relevant key-frames ṽ*_i_*. This video Ṽ instead of V is then transmitted to BS. In this way, it saves considerable amount of energy and bandwidth. For a detail review of our recent video summarization scheme, the readers are referred to study [21].

### 3.2. Dictionary Learning Process

Redundant and sparse representation modeling of data can well describe signals as a linear combination of a few prototype signal atoms from a pre-specified dictionary [22]. Therefore the choice of the dictionary that makes the signals sparse is very important for the success of this redundant and sparse representation model. Aharon *et al.* [33] developed a simple and efficient K-SVD dictionary learning scheme. K-SVD generalizes the idea of K-Mean and uses sequential updating for dictionary [38]. We consider a set of frame patches 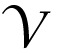 = { 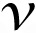
_(1)_, …, 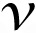
_(n)_}, where the size of each patch is $\sqrt{n}\times \sqrt{n}$ pixels, ordered lexicographically as a column vectors 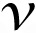
_1_∈ 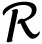
^n^. The K-SVD algorithm learns a dictionary 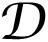
={ 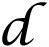
_(1)_, …, 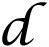
_(m)_}∈ 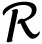
^n^×^m^, 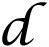
_(i)_ is a prototype signal atom also called filter. The corresponding matrix of sparse representation 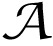
={ 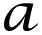
_(1)_, …, 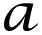
_(n)_}∈ 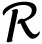
^m^×^n^ of the example set 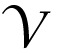 can be recovered by minimizing the following reconstruction error ɛ:

(4) $$\underset{D,A}{\min}\underset{i}{\text{∑}}∥a_{\left(i\right)}{∥}_{0}:∥V-DA{∥}_{\text{F}}^{2}\leq ɛ$$

A similar objective could alternatively be attained by considering the sparse coding problem as a sparsity-constrained:

(5) $$\underset{D,A}{\min}∥V-DA{∥}_{\text{F}}^{2}:\forall i,∥a_{\left(i\right)}{∥}_{0}\leq \text{K}$$

where K is the sparsity level that bounds the number of non-zero entry. 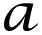
_(*i*)_ are the columns of 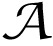 and zero-norm ‖‖_0_ counts the number of non-zero entries in 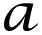
_(*i*)_. The optimization problem posed in (5) can be solved through an alternating manner. In the first stage, the sparse coding is considered, where the dictionary 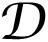 is assumed to be fixed. The problem in (5) can be decoupled to *n* simple sub-problems of the form:

(6) $$\underset{a_{\left(i\right)}}{\min}∥v_{\left(i\right)}-Da_{\left(i\right)}{∥}_{\text{F}}^{2}:\forall i,∥a_{\left(i\right)}{∥}_{0}\leq \text{K}$$

The optimization problem in (6) is combinational and highly non-convex, but any pursuit algorithm can be used to compute the approximate solution. Elad and Aharon [39] and Zeyde *et al.* [32] used OMP to recover the associated matrix of sparse representations over redundant dictionary. The OMP works in greedy style and selects the filter with the highest correlation to the current residual 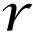 at each step. On selection of a new filter, the observation 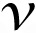
_(*i*)_ is orthogonally projected to the span of all atoms selected previously, the residual 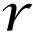 is computed, and the process repeats until the desired sparsity is achieved. The computational cost of the OMP increases while handling large sets of signals, because 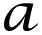
_(*i*)_ or 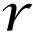 are required explicitly for the atom selection step at each iteration. According to the key-finding, knowing of 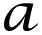
_(*i*)_ or 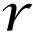 is not needed in each iteration, but only 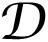
^T^ 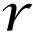 [22, 38]. The idea is therefore to incorporate batch-OMP (BOMP) because it replaces the explicit computation of 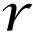 and its multiplication by 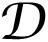
^T^ with a lower-cost computation of 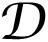
^T^ 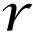. let 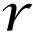
^0^ = 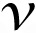, ξ^0^= 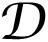
^T^ 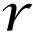
^0^, ξ= 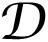
^T^ 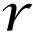, Gram-matrix G= 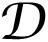
^T^ 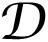, and pseudo-inverse 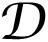
^+^=( 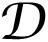
^T^ 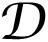)^−**1**^ 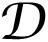
^T^, we can write:

(7) $$\begin{array}{cc} \xi & =D^{\text{T}}(v-D_{\left(\text{I}\right)}{\left(D_{\left(\text{I}\right)}\right)}^{+}v)\\ & =\xi^{0}-{\text{G}}_{\left(\text{I}\right)}{\left({\text{G}}_{\left(\text{I},\text{I}\right)}\right)}^{-1}\xi_{\text{I}}^{0} \end{array}$$

where G_(I)_ denotes the sub-matrix of G containing the columns indexed by (I) while G_(I,I)_ represents the sub-matrix of G containing the rows and columns indexed by (I,I). Equation (7) indicates that if ξ^0^ and G are pre-computed, then, we can compute ξ without explicitly computing 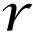 in each iteration. This reduces the total computation time of BOMP. The BOMP is briefly described in Algorithm 1, for detailed study, readers are referred to [22,38].

**Algorithm 1 Batch-OMP**
**1.****Input:** Large set of signals 

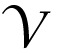
 ={ 

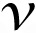
_(1)_, …, 

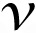
_(n)_}, Dictionary 

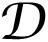
={ 

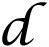
_(1)_, …, 

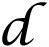
_(m)_}, target sparsity K**2.****Output:** Sparse-representation vector 

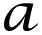
 | 

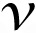
= 

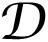


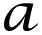
**3.****Init:** I:=( ), 

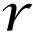
^0^= 

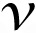
, ξ^0^= 

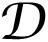
^T^

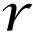
^0^, ξ= 

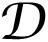
^T^

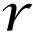
, 

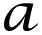
=0, and Gram-matrix G= 

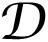
^T^

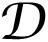
**4.****While** (required sparsity not met) **do**
i)Seek new prototype signal atom: $k=\underset{k}{\text{argmax}}\left|\xi_{k}\right|$ii)I=(I, 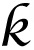)iii)
$a_{\left(\text{I}\right)}={\text{G}}_{\left(\text{I},\text{I}\right)}\xi_{\left(\text{I}\right)}^{0}$iv)ξ = ξ^0^ -G_(I)_ 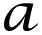
_(I)_**5.****End While**


In the dictionary updating stage, the dictionary 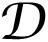 and its corresponding sparse representations 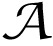
={ 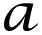
_(1)_, …, 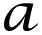
_(n)_} are updated simultaneously by singular value decomposition. Suppose, Dictionary 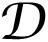 and sparse representations matrix 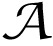 are fixed. For 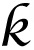
th row of sparse coefficients matrix 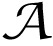 denoted as $a_{\left(k\right)}^{\text{T}}$ associated with 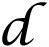
*_
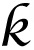
_* column in 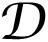, the optimization problem in (5), can be rewritten as:

(8) $$\begin{array}{ll} ∥V-DA{∥}_{\text{F}}^{2} & ={\left\|V‐\underset{j=1}{\text{∑}}d_{\left(j\right)}a_{\left(j\right)}^{\text{T}}\right\|}_{\text{F}}^{2}\\ & ={\left\|\left(V‐\underset{j\neq k}{\text{∑}}d_{\left(j\right)}a_{\left(j\right)}^{\text{T}}\right)-d_{\left(k\right)}a_{\left(k\right)}^{\text{T}}\right\|}_{\text{F}}^{2}\\ & ={\left\|E_{k}-d_{\left(k\right)}a_{\left(k\right)}^{\text{T}}\right\|}_{\text{F}}^{2} \end{array}$$

where 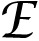
_
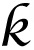
_ is the residual matrix for the $a_{\left(k\right)}^{\text{T}}$. Singular value decomposition is performed of the residual matrix 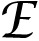
_
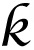
_ to find the optimal 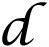
_
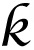
_ and $a_{\left(k\right)}^{\text{T}}$. Singular value decomposition finds the closest rank-1 matrix that approximate 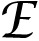
_
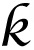
_ and it reduces the error efficiently as defined in (8). Matlab package of [22] and [39] have been used to converge Equations (6) and (8). The learned dictionary 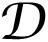 demonstrates basic patterns of the image patches, such as oriented edges, lines and bars instead of raw patches prototypes.

### 3.3. Image Super-Resolution

In image SR, reconstruction constraint and sparsity prior are two important constituents as described by [29]. Reconstruction constraint dictates that the recovered HR frame v must be consistent with LR frame ṽ with-respect-to the image observation model. ṽ is the observed LR version of the corresponding HR frame v_(i)_ which is downsampled and blurred:

(9) $$ṽ={\text{B}}_{\left(\text{d}\right)}\text{v}+\beta$$

where **B_(d)_** is the interpolation operator for downsampling and β is the interpolation error or blurring effect. In sparsity prior, the HR frame v can be well represented as a sparse linear combination of the atoms selected from the dictionary 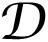 trained from the training images:

(10) $$\begin{array}{ccc} \text{v}\approx DA & \text{where} & \underset{A}{\min}\underset{i}{\text{∑}}{\left\|A\right\|}_{0}:{\left\|ṽ-DA\right\|}_{\text{F}}^{2}\leq ɛ \end{array}$$

The sparse-representation matrix 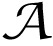 is recovered by representing ṽ over 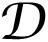 and then using the sparse-representation matrix 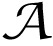, the HR version v is approximated v ≈ **
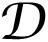

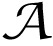
**. The input image ṽ is scaled up by factor ƒ using classic bicubic interpolation **B**_(**up**)_:

(11) $$\bar{\text{v}}={\text{B}}_{\left(\text{up}\right)}ṽ+\beta_{\left(\text{up}\right)}$$

where v̄ is the upscaled LR version and β_(**up**)_ is the blurring effect for bicubic interpolation **B**_(**up**)_. The task is to recover HR image v of high-quality from the blurred LR version v̄ using the problem posed in equation (10). The sparse-representation vector 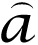
_(i,j)_ is determined for each patches ${\bar{\text{v}}}_{\left(\text{i},\text{j}\right)}^{\left(\text{P}\right)}$ of size $\sqrt{n}\times \sqrt{n}$ extracted from v̄:

(12) $${\overset{⌢}{a}}_{\left(\text{i},\text{j}\right)}=\underset{a}{\text{argmin}}{\left\|a\right\|}_{0}+{\left\|{\bar{\text{v}}}_{\left(\text{i},\text{j}\right)}^{\left(\text{P}\right)}-Da\right\|}_{2}^{2}$$

It works as a sliding-window, operate on each patch at a time. The problem posed in (12) is solved using OOMP. OOMP is a simple modification of the OMP [30]. It updates the residual similar to that of OMP, but detecting the optimal atom at any iteration is different from OMP. Denoting ${\left({\overset{⌢}{A}}_{\left(\text{I},\text{j}\right)}\right)}^{+}={\left({\overset{⌢}{A}}_{\left(\text{I},\text{j}\right)}^{\left(\text{T}\right)}{\overset{⌢}{A}}_{\left(\text{I},\text{j}\right)}\right)}^{-}{\overset{⌢}{A}}_{\left(\text{I},\text{j}\right)}^{\left(\text{T}\right)}$, we can write:

(13) $$\overset{⌢}{a}=\underset{{\overset{⌢}{a}}_{\left(\text{j}\right)}\in \overset{⌢}{A}/{\overset{⌢}{A}}_{\left(\text{I},\text{j}\right)}}{\text{argmax}}\left|{\overset{⌢}{A}}_{\left(\text{I},\text{j}\right)}{\left({\overset{⌢}{A}}_{\left(\text{I},\text{j}\right)}\right)}^{+}{\text{v}}_{\left(\text{i},\text{j}\right)}^{\left(\text{P}\right)}\right|$$

where 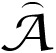
_(I,j)_ denotes the matrix containing all the selected signal atoms index by I except 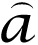. The signal vector is projected onto the subspace spanned by the atoms already selected and under-consideration. The atom having the largest projection is identified as the optimal atom 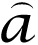. The OOMP does better in terms of seeking a set of high-quality signal v close to the original one, while satisfying the sparsity requirement. Given all detected atoms 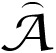 = { 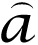
_(1)_,…, 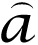
_(k)_}, the final HR image v can be computed by solving the following minimization problem

(14) $$\text{v}=\underset{\text{v}}{\text{argmin}}{\left\|\text{v}-\bar{\text{v}}\right\|}_{2}^{2}+{\left\|\bar{\text{v}}-D\overset{⌢}{A}\right\|}_{2}^{2}$$

The problem posed in (14) can be repeated for each image in the summarized sequence obtained from VPH.

## 4. Experimental Results and Discussion

In order to properly assess the effectiveness of the proposed technique, various sets of experiments were conducted. The images were obtained from various datasets [40-45]for training and evaluation. The images in these databases are taken by cameras consisted of high-quality sensors. High-quality camera-sensor produces an image containing more effective representations of different scenes. These images contain a variety of structures that are most appropriate for dictionary training and evaluation. These images make able the dictionary to captures much richer types of statistical dependencies. A dictionary containing effective image representation can help SR scheme to produce HR output of high-quality. Moreover, these standard datasets also play a role of benchmark, therefore we considered these datasets for evaluation. The dictionary was trained from a set of 10^6^ examples of patches of size 9×9 randomly sampled from images obtained from various data-sets. Unless explicitly mentioned, the size of the dictionary was kept 2^11^ in all experiments. The proposed SR algorithm was tested on a Core i5 desktop system @3.40G with 8 GB memory. This desktop system was pretended as a BS in our experiment. [46] and [30] experimentally proved that OOMP is much better than OMP in terms of sparsity and true atom detection. Therefore, we did not repeat the evaluation previously done in [46] and [30] and rather concentrated our quantitative analysis on the SR image of the proposed scheme with other state-of-the-art techniques.

### 4.1. Quantitative Evaluation

The quantitative assessment was conducted by comparing the proposed scheme with other state-of-the-art techniques including simple bicubic, Sajjad *et al.* [14] (MK-SR) and Zeyde *et al.* [32] (SI-SR). For this purpose, the images compressed by jpeg2000 and their corresponding reference images were downloaded from the Live database [41] as shown in Table 1. The compressed images were down-sampled by a factor 4 and scaled up to the original size using bicubic, MK-SR, SI-SR and the proposed SR scheme. The peak signal to noise ratio (PSNR) and structure similarity index metric (SSIM) [42] were computed for each technique to assess the quality of the reconstructed signal. PSNR computes the ratio between the strength of the maximum achievable power of the reconstructed signal and the strength of the corrupted noisy signal. The signal indicates the original information and noise represents the error added during the reconstruction process. A higher value of the PSNR shows the effectiveness of the SR scheme:

(15) $$\text{PSNR}=20\times {\text{log}}_{10}\left(\overset{f_{\text{max}}}{\;}/\underset{\sqrt{\text{MSE}}}{\;}\right)$$

where *f_max_* is the maximum fluctuation of the original image and $\text{MSE}=\underset{\text{x}=1,\text{y}=1}{\text{∑}}{\text{v}(\text{x},\text{y})-\hat{\text{v}}(\text{x},\text{y})}^{2}/\text{M}\times \text{N}$ is the mean squared error. Here v represent the reconstructed HR frame and v̂ is the original reference frame. M**×**N is the size of the underlying frame. Table 2 indicates that the quality of the signal reconstructed by the proposed scheme is better than other state-of-the-art techniques. Both SI-SR and the proposed scheme conduct SR and denoising simultaneously, however bicubic and MK-SR can only increase the resolutions of the underlying image and cannot clean the noisy signal. Therefore, their PSNR's score is less than SI-SR and the proposed technique. The PSNR score of the proposed scheme is comparatively better than SI-SR due the way it learns the dictionary and determines sparse-representation coefficients.

Wang *et al.* [42] proved that MSE does inaccurate assessment in various situations. Therefore, to confirm the effectiveness of the proposed scheme, the SSIM is used to evaluate the quality of the reconstructed frames at BS. SSIM has the capability to capture the local statistical features of the underlying signal and very sensitive to various types of distortion during SR. It focuses on the distortion of the geometrical structures of the original reference image v̂. Therefore, SSIM is more effective in evaluating the spatial-temporal inconsistency of the output SR v:

(16) $$\text{SSIM}=\frac{\left(2\mu_{\overset{⌢}{\text{V}}}\mu_{\text{V}}+{\text{const}}_{1})(2\sigma_{\overset{⌢}{\text{V}}\text{V}}+{\text{const}}_{2}\right)}{\left(\mu_{\overset{⌢}{\text{V}}}^{2}+\mu_{\text{V}}^{2}+{\text{const}}_{1})(\sigma_{\overset{⌢}{\text{V}}}^{2}+\sigma_{\text{V}}^{2}+{\text{const}}_{2}\right)}$$

where σ represent the variance and *const_1_* and *const_2_* are constant to stabilize the division with a weak denominator. *μ*_v̂_, *μ*_v_, and *σ*_v̂v_ are the local statistics that are computed within an 8×8 window locally and move pixel by pixel over the entire image like a sliding window. The mean SSIM (MSSIM) is comput ed to get a single overall quality measure for the evaluation of the corresponding image. The value near to one indicates high quality of the underlying image. Table 3 contains MSSIM score for the proposed scheme and other mentioned state-of-the-art schemes. Bicubic and MK-SR have the lowest MSSIM score because of not differentiating between noisy and clean signals. SI-SR and the proposed scores are better than Bicubic and MK-SR but the proposed scheme has a slightly high score than SI-SR. The scores listed in Table 3 justify the superiority of the sparse-coding and dictionary-learning methods used by the proposed technique.

As indicated in the framework (Figure 1), VPH receives sequences of frames from visual sensors, containing redundant information. It is largely agreed that only relevant and non-redundant frames should be streamed to BS. At VPH, we applied our recent video summarization technique to extract key-frames.

In Table 4, two videos downloaded from datasets mentioned earlier were summarized. Video#1, consist of 2,310 frames and video#2 consists of 3,700 frames. On summarization, we got 10 and 13 frames for video#1and video#2 respectively. Summarized and compressed frames are forwarded to BS where, the proposed SR scheme was applied to get the HR version of the received frames.

**Figure 2.**
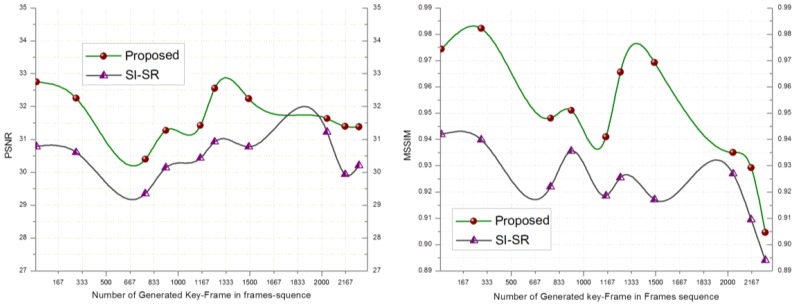
PSNR and MSSIM computed for key-frames of the Video#1.

**Figure 3.**
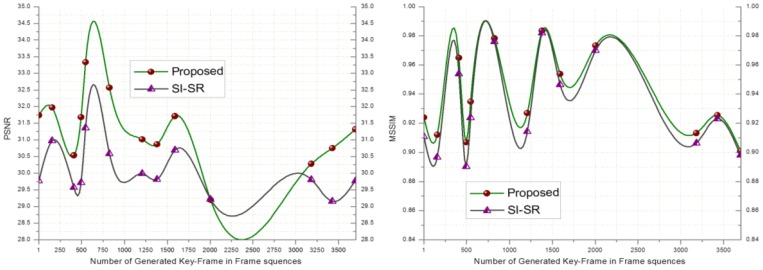
PSNR and MSSIM computed for key-frames of the video#2.

In order to evaluate the performance of the proposed SR scheme, the received key-frames at the BS are downsampled by a factor of 4 and scaled up to the original size using SI-SR and the proposed SR scheme. Bicubic and MK-SR were not included in this experiment because of lacking the denoising property. Figure 2 shows that the PSNR and MSSIM score of the proposed scheme goes higher than SI-SR and a similar result can be seen in Figure 3.

### 4.2. Energy Consumption Analysis

In this subsection, energy consumption analysis is briefly presented to highlight the energy-saving aspect of the proposed framework. We followed the experimental procedure of [47] and [48]. The energy consumption was examined using Tmote sky motes platform. We used the same specification for Tmote platform as used by [47]. Therefore, readers are referred to [47] and [48] for more details. We took assumptions such as: (1) the communication link is contention-free; (2) VPH does not fail during frame transmission; (3) all the packets are sent through a reliable path. We also ignored the overhead energy consumptions. Four videos were taken from the databases mentioned earlier. The frame size of each video was adjusted to the size 300 × 300 bytes using classic bicubic algorithms. Each frame was in RGB color format, hence, the total size became 300 × 300 × 3 bytes. The expected total transmission energy 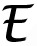
_t_ of frame transmission is:

(17) $$E_{t}=ne_{\text{p}}$$

where 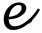
_p_ is the required energy to transmit one data packet and *n* is the number of total packets to be transmitted. According to [47], in Tmote platform the energy 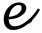
_p_ required to transmit a packet is 88.48 μJ, where the packet consists of 8 × 8 macro-block with 2 bytes offset information and 13 bytes 802.15.4 header. Table 5 shows that an efficient summarization can reduce the bandwidth consumption up to a greater extent. Non-redundant data also facilitate the analyst at BS to process relevant data only, and hence, improve the decision and diagnosis process of the surveillance system.

Moreover, the energy-saving aspect of the proposed framework is very obvious and simple. Low-quality sensors are used to generate LR frames sequence that consumes less energy in capturing and transmission than high-quality sensors. However, High-quality sensors produce HR frames, consume high-energy and bandwidth while transmitting from camera sensors to VPH. According to the literature, HR images are not usually preferred at the camera-sensor end but they are favored at BS for conclusive analysis and diagnosis in most surveillance applications [3,11,47].

### 4.3. Subjective Evaluation

**Figure 4.**
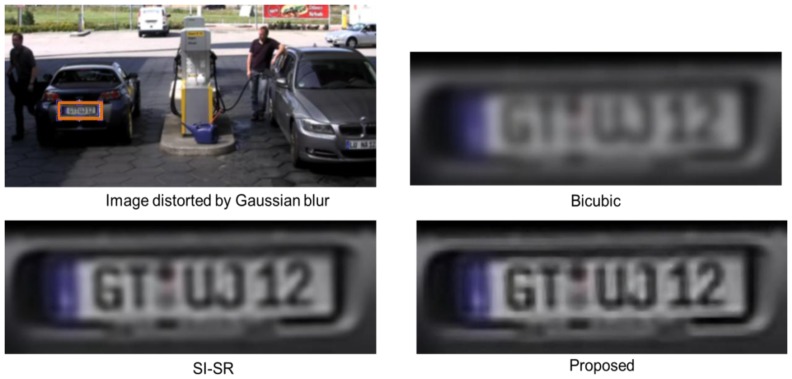
Visual quality evaluation of the proposed scheme on images distorted by Gaussian noise (σ = 3, kernel size = 5).

**Figure 5.**
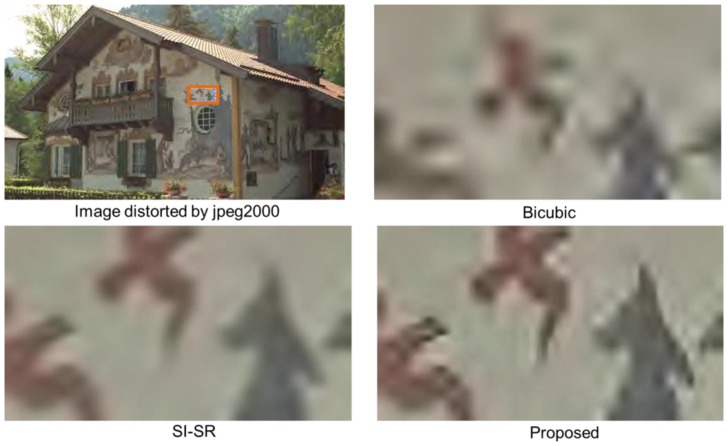
Visual quality assessment of the proposed scheme on image distorted by jpeg2000 (bitrate = 0.41508).

**Figure 6.**
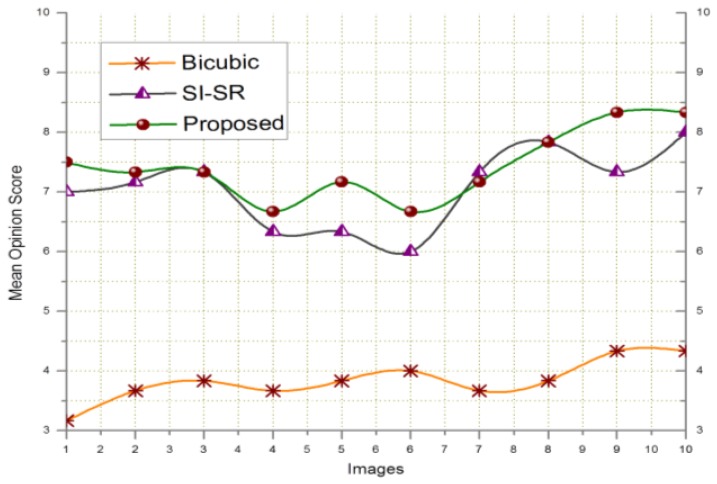
Subjective evaluation of the proposed and other schemes using MOS.

The visual quality of the proposed technique was evaluated by comparing it with bicubic and SI-SR schemes. In this experiment, we used YCbCr color space for color image reconstruction. The advantage of YCbCr color space is based on the characteristics of the human visual perception (that is, related to the visual cortex) where Y component describes the luma i.e., the brightness, Cb is the blue difference (B-Y) and Cr is the red difference (R-Y). The human eye is more sensitive for the luma as compared to chrominance information. Our SR technique gives less importance to the color information because their signals are smoother than the luma's signals. Therefore, Cb and Cr components of the input image were scaled up using classic bicubic interpolation, while Y component was reconstructed by the proposed SR scheme. However, the proposed SR scheme can be easily extended to other types of color space e.g., RGB color space. For visual evaluation, we considered seven parameters of Wittman *et al.* [49] as a touchstone to assess the visual quality of the HR output. According to [49], an SR algorithm must preserve the: (1) edges; (2) tex0ture; (3) contrast invariance and (4) geometric invariance of the input image. It must also retain the characteristics of: (5) anti-aliasing, (6) denoising, and (7) preventing the input image from over-smoothing. We took two images from public databases for this purpose. In Figure 4, a patch of 26 × 70 pixels was cropped from the image highlighted by the orange-color rectangle. This cropped patch was scaled up 4× using bicubic, SI-SR, and the proposed schemes. The image was distorted by Gaussian blur (sigma σ = 3, kernel size= 5 × 5). Bicubic scheme shows worsen visual quality, because it has no denoising property. The visual result of the proposed scheme and SI-SR can be compared and the result shows that the proposed technique obeyed the seven parameters of [49] up to a greater extent. In Figure 5 the image was degraded using jpeg2000 compression (bitrate = 0.41508). The same procedure was repeated as in Figure 4. The size of the cropped patch is 30 × 60 pixels. The proposed technique again presented comparatively better visual quality than SI-SR. The HR output was also assessed by using a mean opinion score (MOS) based on subjective user studies base criterion. The same subjective evaluation was used before by [21] and [14]. For this purpose, the service of ten students was acquired (five females and five males). These students were working in various Labs of the Digital Content Department in the vicinity of our lab. The importance of the seven parameters was described and the students were trained for one hour. Then they were given both reference images and the corresponding reconstructed images (by our method and other mentioned schemes) for evaluation. They were asked to rate the quality of the reconstructed images (that is, scale from 1 (bad quality) to 10 (good quality)). Figure 6 presents the subjective evaluation using MOS for ten images downloaded from public databases. The subjective assessment based on direct opinion of the students, shows that the proposed scheme attained the highest MOS.

### 4.4. Limitation of the Proposed Scheme

The proposed scheme conducts SR and denoising simultaneously. To evaluate this feature of the proposed SR scheme, various images were distorted using jpeg2000 compression and Gaussian blur. These distorted images were downsampled by factor two and were reconstructed to the original size using the proposed SR scheme. The PSNR and MSSIM were computed to evaluate the quality of the reconstruction process of the proposed scheme. The results for both PSNR and MSSIM are given in Tables 2 and 3. The proposed SR scheme was also evaluated qualitatively as discussed in Section 4.3. While evaluating the proposed scheme, we found that there are some limitations, where the proposed SR scheme does not work very well and it cannot completely reconstruct and clean the underlying image. Sometimes, the surrounding environment and unpredictable weather conditions (e.g., fog, rain, strong wind, malfunctioning-sensors and image occlusion) can severely degrade the quality of the frames sequence captured by the visual sensor. We observed that the quality of the proposed SR scheme gets worsen when the degradation crosses a particular threshold i.e., degradation is inversely proportion to the quality of the proposed SR scheme. To handle these limitations, in future we have intention to include fusion schemes and dictionary learning from multi-view images (images of the same scene taken by different camera sensors from different views).

## 5. Example-Based Study

In this section, we present a descriptive analysis of a prospective example related to VSNs. To make the analysis unbiased, we downloaded four videos from the ETISEO database [50]. These videos are of the same scene taken from four different views. The videos are mentioned with their respective details in Tables 6 and 7, where in video-name C represents camera and numeric value with C such as C1 represent camera number. In this particular example, we assumed that VPH receive and buffer the videos from four cameras and process them one by one. For energy analysis, we considered the same assumptions that were taken in Section 4.2. Our recent key-frame extraction scheme [21] was applied at the VPH to extract key-frames. Table 6 shows the key-frames extracted for each video. The irrelevant and redundant frames were trashed and only key-frames were forwarded to the BS. In this example based study, jpeg2000 compression technique was applied to each key-frame at the VPH before forwarding them to the BS, which further degrades the quality of the key-frames. This degradation also reflects a slightly bad weather condition. The size of each key-frame was adjusted to the size 300 × 300 bytes using classic bicubic algorithms. Each frame was in RGB color format, hence, the total size became 300 × 300 × 3 bytes. The expected total transmission energy for key-frames and full shot of each video was computed using formula mentioned in Equation (17). Table 7 shows the energy consumption evaluation. The total energy consumption of key-frames for four video is 10,825,176 μJ while this is for four full-shot video is 1.3345 × 10^9^ μJ. The difference is 1.3236 × 10^9^ μJ, i.e., energy is being saved in this difference. At the BS, LR key-frames which were not suitable for any analysis were received. Therefore, the proposed SR scheme on each LR key-frame was applied to reconstruct its corresponding HR version of high-quality. To evaluate the quality of the reconstructed key-frames, we computed the PSNR and MSSIM for each key frame using the formulas mentioned in Equations (15) and (16), respectively. The averaged value PSNR and MSSIM of key-frames for each shot is listed in Table 7.

The reconstructed HR version of each key-frame had high-quality and was most suitable for various analysis and diagnosis process. Moreover, the video in summarized (i.e., key frames) form can also help users to quickly locate a semantically relevant position in frames sequence captured by the visual sensors.

## 6. Conclusions

This paper has presented a novel framework toward SR for LR key-frames extracted from video sequences captured by camera nodes. VPHs gather video sequences in large amounts from camera sensors. Only relevant data and non-redundant video frames should be streamed to the BS in order to minimize the bandwidth consumption. For this purpose, we have incorporated our recent efficient video summarization technique at the VPH to extract key-frames from the frames sequence collected from visual sensor nodes. An effective SR scheme is applied at the BS on the received LR key-frames to produce a HR output, which is more suitable for analysis in various surveillance applications. The dictionary in the proposed scheme learns redundant image representations from large set of training signals using batch-OMP (i.e., can be hardly achieved using simple OMP). The detection of true sparsity by OOMP makes the SR process more robust toward denoising and reconstruction of the underlying key-frames. Various quantitative and qualitative evaluations validate the effectiveness of the proposed scheme. In the future, the role of the redundant dictionary can be extended to other image processing operations in VSNs. Fusion of multi-view images can be incorporated to cope with the limitations of the proposed system. We are also enthusiastic to optimize onboard processing keeping in mind the trade-off between energy consumption and computational complexity.

## Figures and Tables

**Table 1.** Name of images with their corresponding bit-rate (compressed by jpeg2000).

**Image. No**	**Image Name**	**Bit-rate (jpeg2000)**
**1**	Bike	0.6957
**2**	Building	0.72182
**3**	Dancers	0.87657
**4**	Flower-Sonih	0.94912
**5**	Painted-House	0.78388
**6**	Parrots	0.68851
**7**	Sailing Boat	0.59673
**8**	Statue	0.74761
**9**	Student-Sculpture	1.0136
**10**	Woman	0.59812

**Table 2.** PSNR computed for compressed images mentioned in Table 1.

**Image. No**	**Bicubic**	**MK-SR**	**SI-SR**	**Proposed**
**1**	22.38	24.69	27.83	29.48
**2**	18.92	20.73	24.76	27.75
**3**	19.54	21.18	25.76	27.78
**4**	19.13	21.06	24.02	26.58
**5**	23.16	23.94	28.11	29.44
**6**	27.68	28.85	37.67	39.36
**7**	25.45	27.02	30.53	32.09
**8**	25.90	26.21	33.22	34.15
**9**	20.01	23.36	24.01	24.91
**10**	23.66	25.47	28.40	29.68

**Table 3.** MSSIM computed for compressed images mentioned in Table 1.

**Image. No**	**Bicubic**	**MK-SR**	**SI-SR**	**Proposed**
**1**	0.708	0.715	0.912	0.940
**2**	0.682	0.698	0.909	0.926
**3**	0.743	0.744	0.877	0.883
**4**	0.706	0.722	0.924	0.936
**5**	0.746	0.756	0.929	0.935
**6**	0.662	0.664	0.968	0.977
**7**	0.805	0.818	0.904	0.908
**8**	0.719	0.720	0.960	0.985
**9**	0.734	0.741	0.898	0.900
**10**	0.671	0.675	0.922	0.949

**Table 4.** Key-Frames that are extracted at VPH.

**Video. No**	**Generated key-Frames**												
1	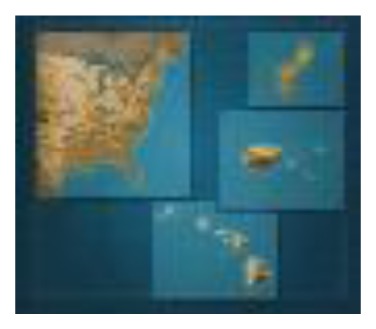	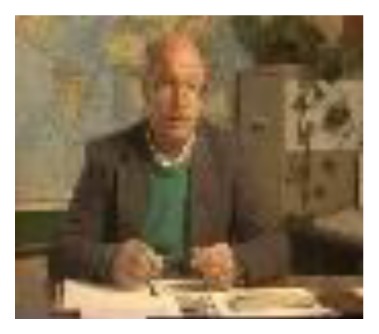	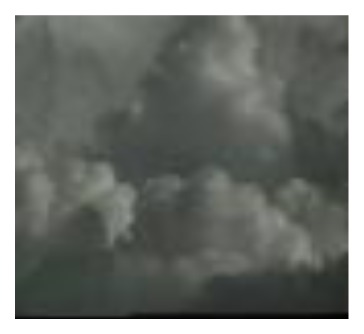	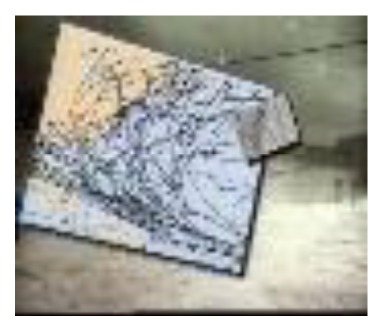	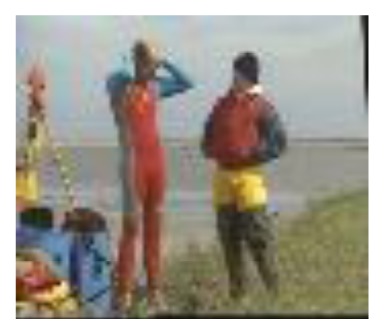	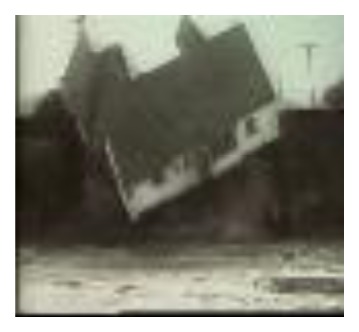	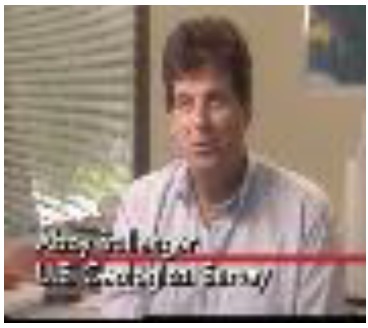	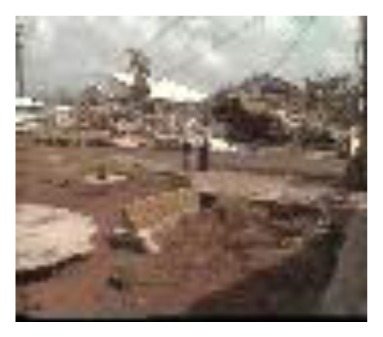	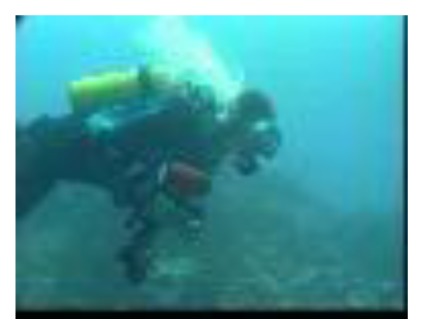	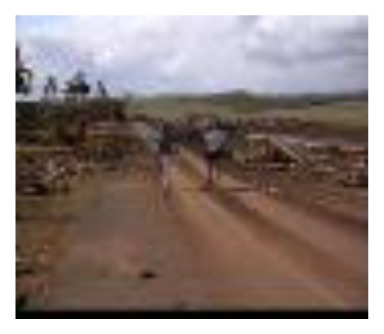			
Frame#	16	292	773	916	1156	1256	1492	2037	2163	2260			

2	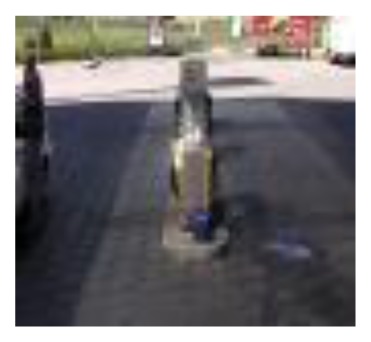	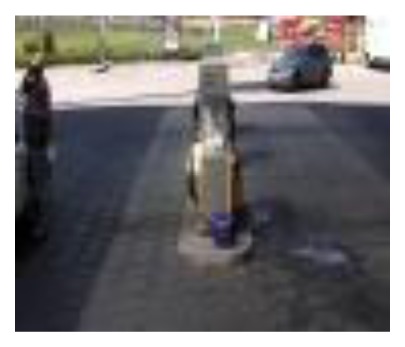	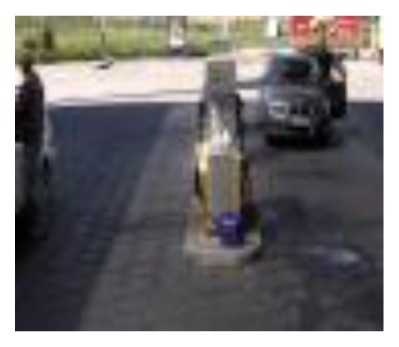	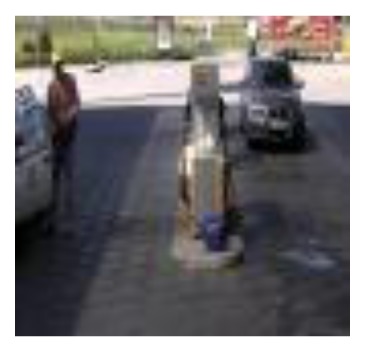	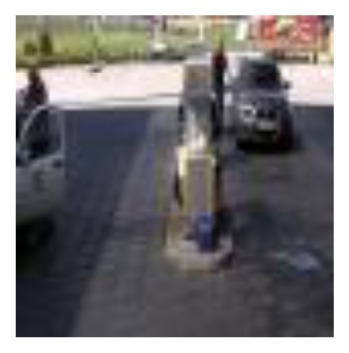	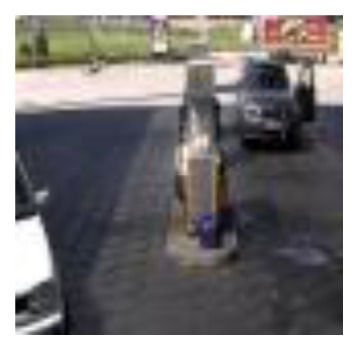	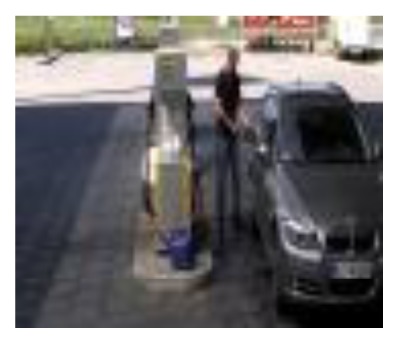	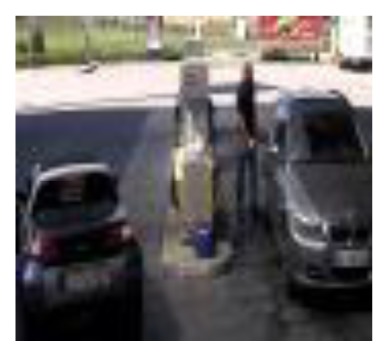	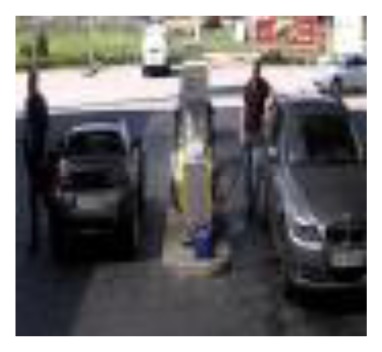	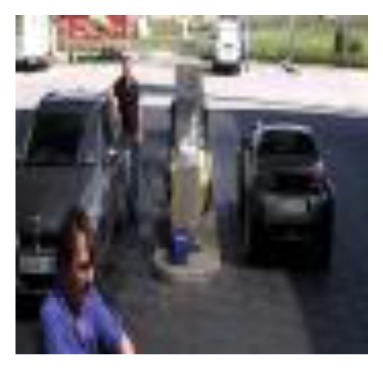	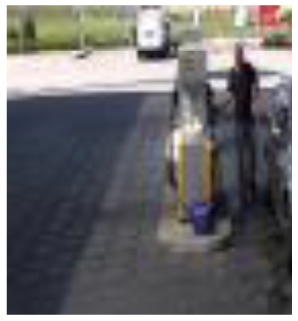	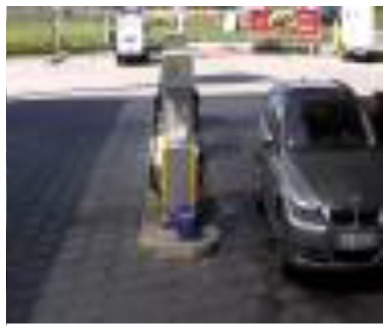	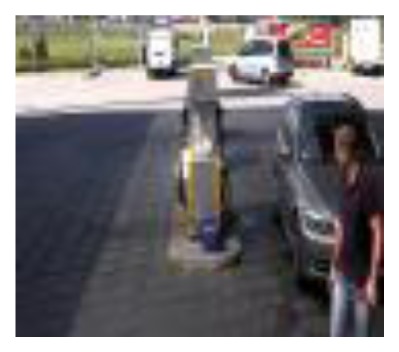
Frame#	1	157	410	494	546	824	1208	1380	1590	2005	3181	3427	3697

**Table 5.** Transmission energy analysis for four videos.

**Video**		**Number of frames**	**Total Number of Packets**	**Energy Required (μJ)**
1	Full shot summarized	2,310	9,745,890	862,316,347.20
		10	42,190	3,732,971.20
2	Full shot summarized	3,700	15,610,300	1,381,199,344.00
		13	54,847	4,852,862.56
3	Full shot summarized	1,600	6,750,400	597,275,392
		7	29,533	2,613,079.84
4	Full shot summarized	2,500	10,547,500	933,242,800
		11	46,409	4,106,268.32

**Table 6.** Key-frames that are extracted at the VPH in our case study.

**Video Name**	**Generated key-Frames**							
ETI-VS2-BE-19-C1	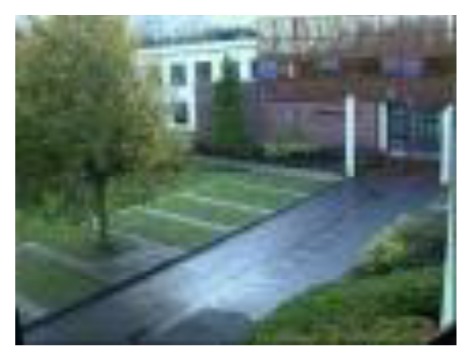	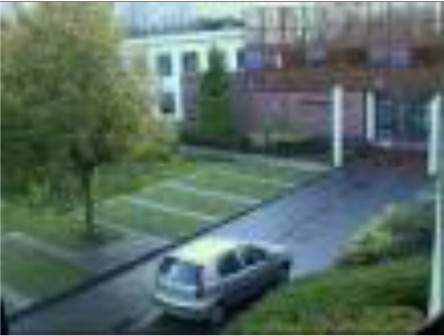	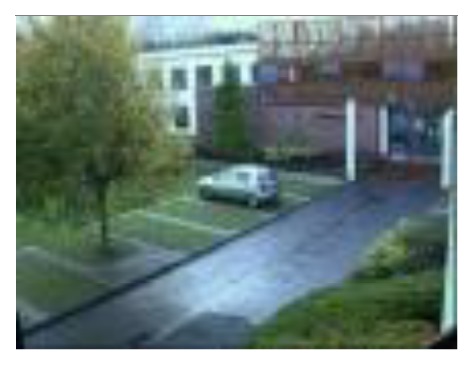	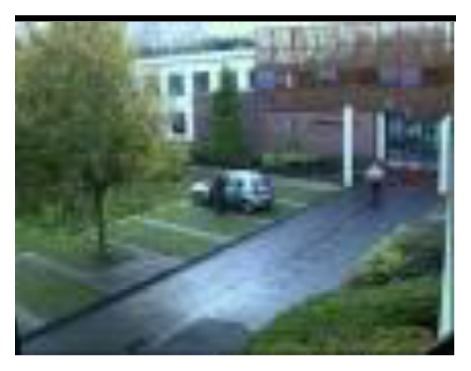	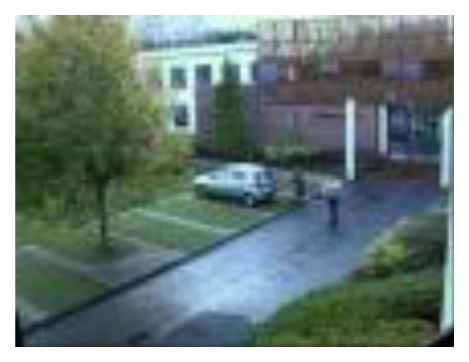	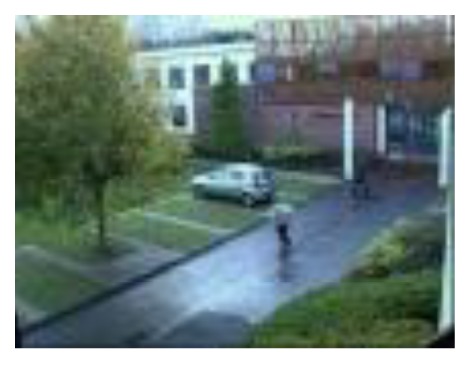	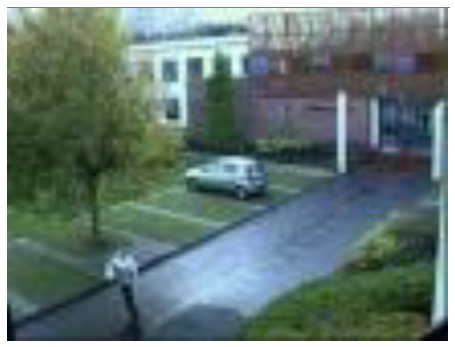	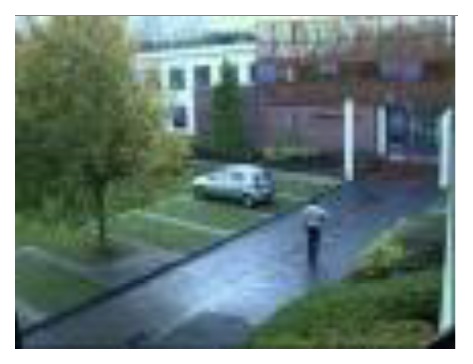
Frame#	1	118	257	446	516	570	662	991

ETI-VS2-BE-19-C2	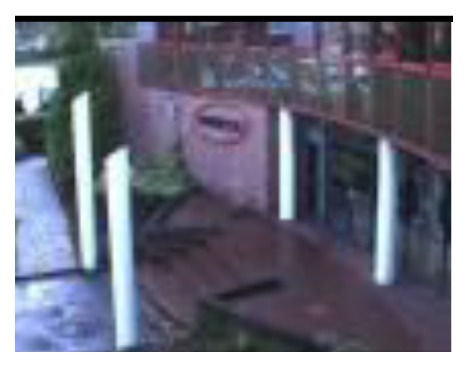	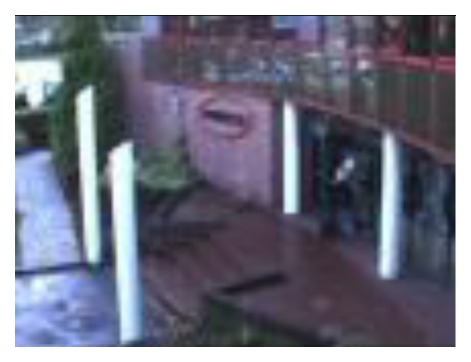	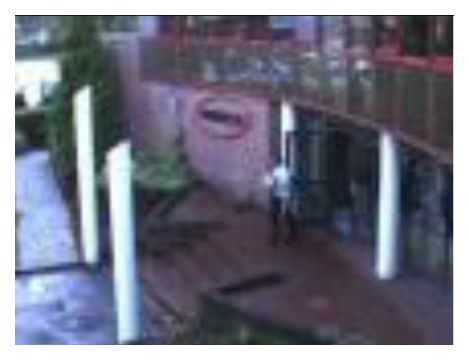	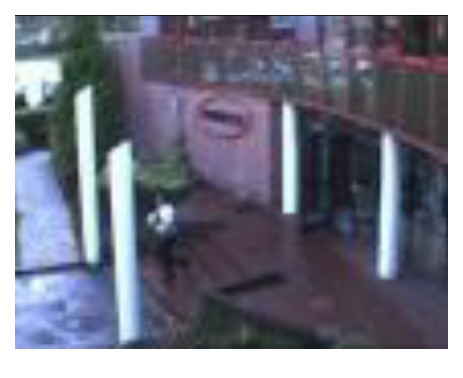	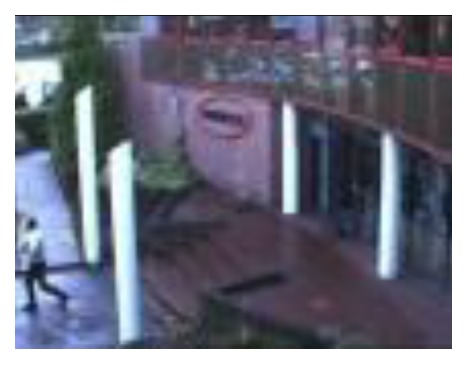	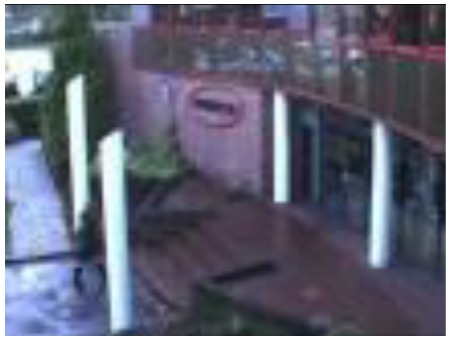	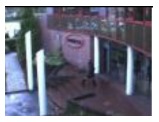	
Frame #	1	53	100	157	207	376	441	

ETI-VS2-BE-19-C3	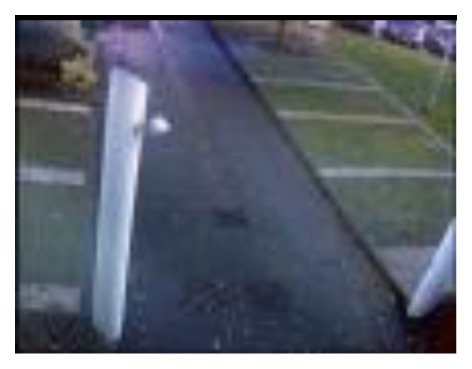	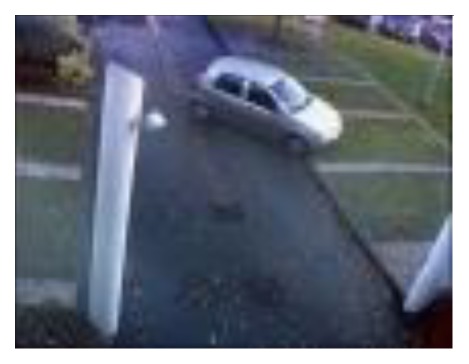	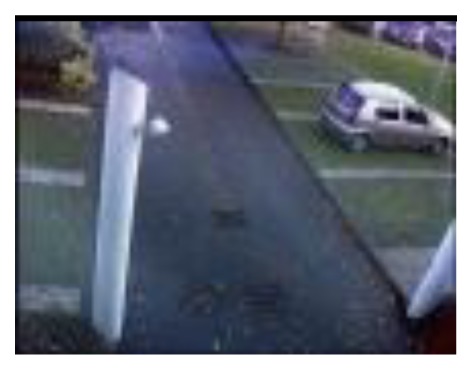	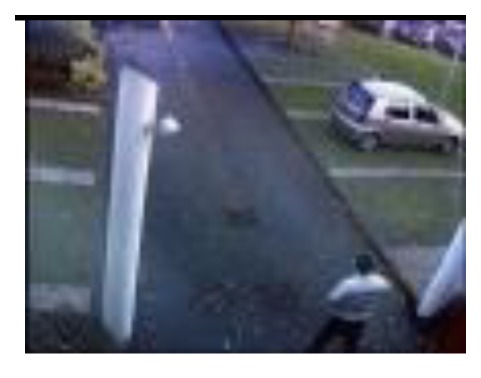	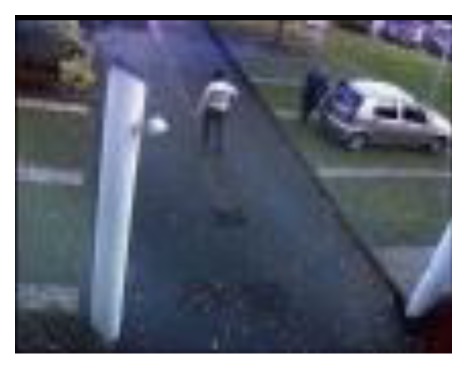	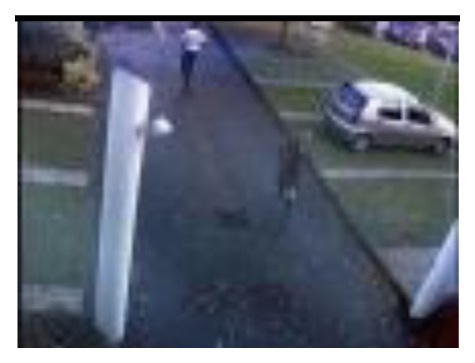	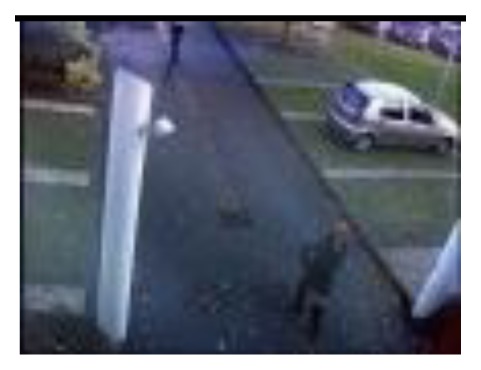	
Frame#	10	171	270	367	471	534	577	

ETI-VS2-BE-19-C4	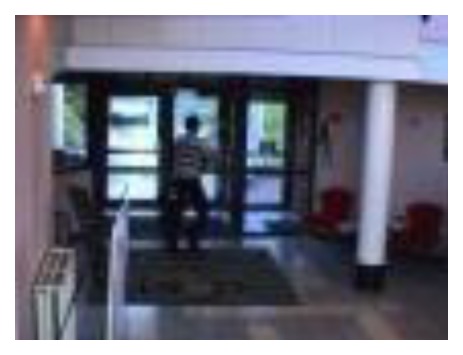	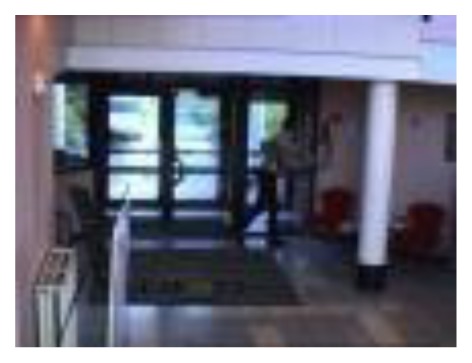	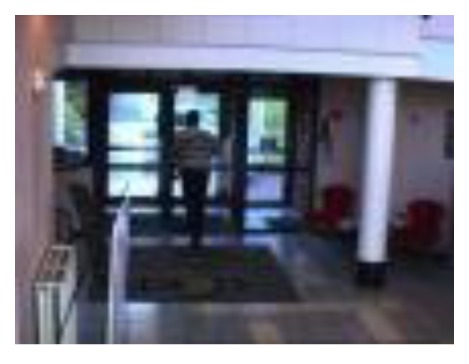	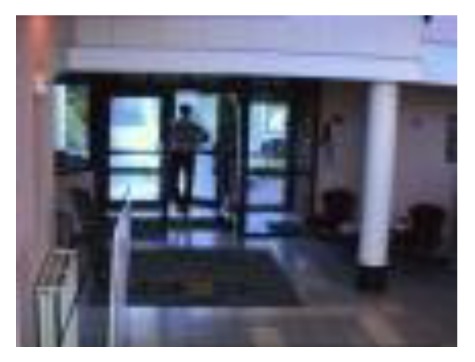	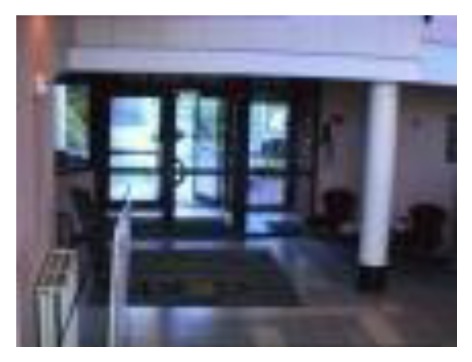	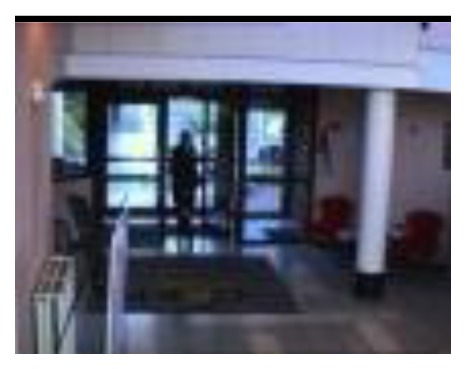	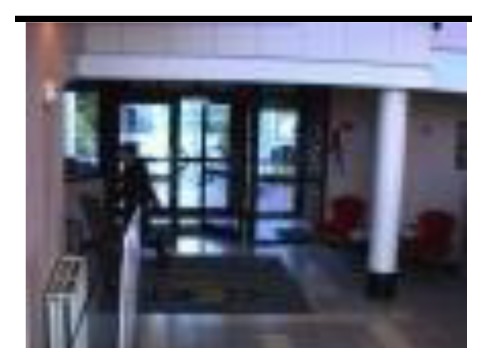	
Frame #	1	39	92	176	279	776	814	

**Table 7.** Transmission energy and Quantitative analysis for four videos of the same scene.

**Video**			**Energy analysis**		**Quantitative analysis (of Key-frames)**	

Video name	Number of frames		Total number of packets	Energy Required (μJ)	PSNR	MSSIM
ETI-VS2-BE-19-C1	Full-Shot Key-frames	1025	4324219	382606898		
		8	33750	2986200	31.1551	0.93153
ETI-VS2-BE-19-C2	Full-Shot Key-frames	875	3691407	326615692		
		7	29532	2612992	28.7285	0.90427
ETI-VS2-BE-19-C3	Full-Shot Key-frames	725	3058594	270624398		
		7	29532	2612992	33.3911	0.94738
ETI-VS2-BE-19-C4	Full-Shot	950	4007813	354611295	27.4693	0.89188
